# 226. Effectiveness of Maternal Influenza Vaccination during Pregnancy against Medically Attended Influenza among Infants <6 Months of Age, New Vaccine Surveillance Network (NVSN), 2016-2017 through 2024-2025 Influenza Seasons

**DOI:** 10.1093/ofid/ofaf695.009

**Published:** 2026-01-11

**Authors:** Savanah Russ, Leila C Sahni, Julie A Boom, Geoffrey A Weinberg, Peter G Szilagyi, Natasha B Halasa, Laura S Stewart, Janet A Englund, Eileen J Klein, Mary A Staat, Elizabeth P Schlaudecker, Jennifer E Schuster, Rangaraj Selvarangan, Marian G Michaels, John Williams, Ayzsa Tannis, Heidi L Moline, Flor M Munoz, Sascha R Ellington, Samantha M Olson

**Affiliations:** US CDC, Atlanta, Georgia; Baylor College of Medicine and Texas Children's Hospital, Houston, Texas; Baylor College of Medicine, Houston, Texas; University of Rochester Sch Med & Dent, Rochester, New York; UCLA, Los Angeles, California; Vanderbilt University Medical Center, Nashville, TN; Vanderbilt University School of Medicine, Nashville, Tennessee; Seattle Children’s Hospital/Univ. Washington, Seattle, Washington; Seattle Children's Hospital and University of Washington School of Medicine, Seatte, Washington; Cincinnati Children's Hospital Medical Center, Park Hills, Kentucky; Cincinnati Children's Hospital Medical Center, Park Hills, Kentucky; Children's Mercy Kansas City, Kansas City, MO; Children’s Mercy Hospital, Kansas City, Missouri; University of Pittsburgh/ CHP, Pittsburgh, Pennsylvania; University of Wisconsin, Madison, Wisconsin; Centers for Disease Control and Prevention, Atlanta, Georgia; US-CDC, Atlanta, Georgia; Baylor College of Medicine Houston, Dallas, Texas; COVID-19 Response, Centers for Disease Control and Prevention, Atlanta, Georgia; Centers for Disease Control and Prevention, Atlanta, Georgia

## Abstract

**Background:**

The 2024-2025 influenza season was a high severity season for infants and children that resulted in the most pediatric deaths since the 2009 H1N1 pandemic. Maternal influenza vaccination during pregnancy is effective at preventing influenza illness in pregnant women and infants, has a strong safety record, and is recommended at any time during pregnancy. We assessed U.S. maternal influenza vaccine effectiveness (VE) against influenza illness among infants < 6 months, for whom there is no licensed influenza vaccine.

**Figure:**
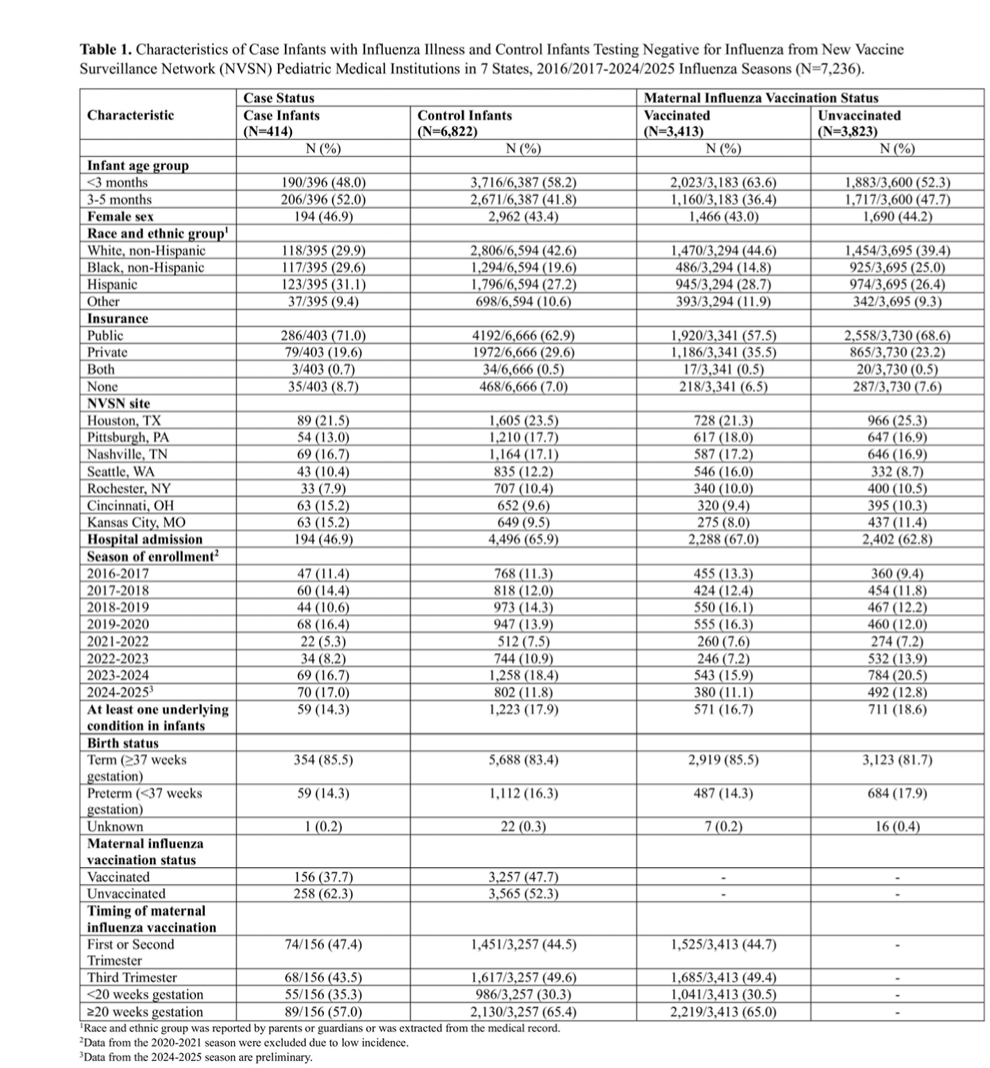

**Figure:**
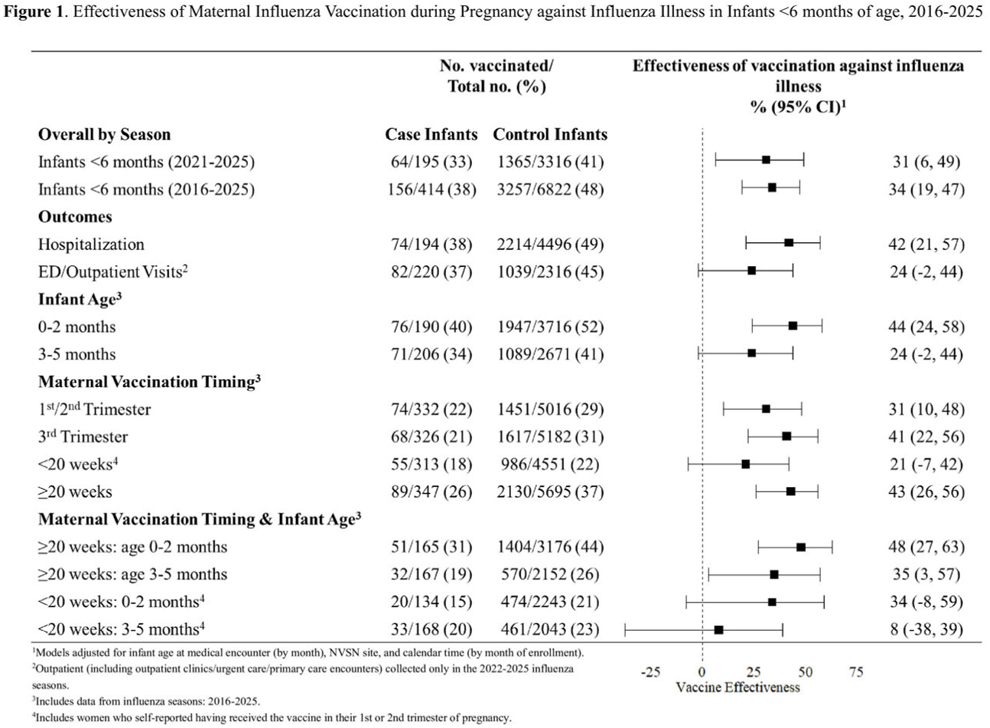

**Methods:**

We used a test-negative design to assess maternal influenza VE against laboratory-confirmed influenza illness among infants < 6 months with acute respiratory illness enrolled at seven pediatric medical institutions from 2016-2025. Influenza testing included molecular research and clinical testing results. Vaccination status was collected via state immunization information systems, providers, or self-report. VE was estimated by comparing the odds of maternal influenza vaccination ≥ 14 days prior to birth in case infants with influenza compared to control infants with non-influenza illness. VE was estimated overall, by medical setting, infant age, and timing of vaccination in pregnancy.

**Results:**

We included 7236 infants; 414 cases and 6822 controls, of which 37% and 47% were born to mothers vaccinated during pregnancy, respectively (Table 1). VE was 34% (95% confidence interval, CI: 19-47%) overall and 42% (95% CI: 21%-57%) against hospitalization (Figure 1).VE was significant for infants born to mothers vaccinated ≥ 20 weeks gestation aged 0-2 (48%; 95% CI: 27%-63%) and 3-5 months (35%; 95% CI: 3%-57%) at encounter. VE was not significant for infants born to mothers vaccinated < 20 weeks gestation aged 0-2 and 3-5 months.

**Conclusion:**

Maternal influenza vaccination overall significantly reduced the odds of influenza illness among infants < 6 months of age, including in the high severity 2024-2025 season. Despite this, maternal influenza vaccination has recently declined, including in our study with less than half of infants born to mothers vaccinated during pregnancy. Efforts are needed to increase influenza vaccination uptake and better understand the optimal timing of influenza vaccination in pregnancy for both mom and baby.

**Disclosures:**

All Authors: No reported disclosures

